# Flavonoid Ampelopsin Inhibits the Growth and Metastasis of Prostate Cancer In Vitro and in Mice

**DOI:** 10.1371/journal.pone.0038802

**Published:** 2012-06-05

**Authors:** Feng Ni, Yi Gong, Linglin Li, Hamid M. Abdolmaleky, Jin-Rong Zhou

**Affiliations:** 1 Department of Pharmaceuticals, Fujian Health College, Fuzhou, Fujian, People's Republic of China; 2 Nutrition/Metabolism Laboratory, Department of Surgery, Beth Israel-Deaconess Medical Center, Harvard Medical School, Boston, Massachusetts, United States of America; The University of Kansas Medical Center, United States of America

## Abstract

The objective of this study was to evaluate the chemopreventive effect of a novel flavonoid, ampelopsin (AMP) on the growth and metastasis of prostate cancer cells. AMP showed the more potent activity in inhibiting the proliferation of androgen-sensitive LNCaP and, to less extent, androgen-independent PC-3 human prostate cancer cell lines in vitro, primarily by induction of apoptosis associated with down-regulation of bcl-2. On the other hand, AMP showed much less activity in inhibiting the proliferation of normal prostate epithelial cells than that of prostate cancer cell lines. AMP also inhibited the migration and invasion of PC-3 cells in vitro associated with down-regulation of CXCR4 expression. In the animal study using an orthotopic prostate tumor model, AMP (150 and 300 mg/kg body weight) inhibited the growth of PC-3 tumors and lymph node and lung metastases in a dose-dependent manner. Compared to the control mice, mice treated with AMP at 300 mg/kg BW had reduced final tumor weight by 49.2% (P<0.05), lymph node metastases by 54.5% (P = 0.3) and lung metastases by 93% (P<0.05), but had no apparent alteration on food intake or body weight. The in vivo anti-growth and anti-metastasis activities of AMP were associated with induction of apoptosis and inhibition of proliferation of prostate cancer cells, reduction of prostate tumor angiogenesis, and reduction of CXCR4 expression. Our results provide supporting evidence to warrant further investigation to develop AMP as a novel efficacious and safe candidate agent against progression and metastasis of prostate cancer.

## Introduction

Prostate cancer is the most common invasive malignancy and the second-leading cause of cancer death in men in the U.S. It is estimated that 241,740 new cases of prostate cancer would be diagnosed and about 28,170 men would die of prostate cancer in the United States in 2012 [1]. Current therapeutic modalities for prostate cancer usually have variable effectiveness, develop metastasis and drug-resistance, and have high toxicity to normal tissues. Therefore, the searching for effective regimens with moderate adverse effects for the chemopreventive intervention of prostate cancer remains the top priority in prostate cancer research.

Bioactive components in botanicals and herbal medicines may provide effective and safe candidates for chemoprevention and/or therapy of cancer. Classical Chinese medical texts contain extensive empirical records of botanical therapies used to treat patients with cancer and cancer-related systems. However, most of these candidate botanical formulas are recommended based on clinical observations only. Moreover, these clinical observations were almost always from uncontrolled, observational studies or anecdotal case reports. Until very recently, there has been limited effort to develop preclinical, mechanistic, scientific evaluation of botanical herbal products as a prerequisite for human clinical studies.

The Chinese herb *Ampelopsis grossedentata* is widely distributed in South China and is used to treat cold and tinea corporis. It contains a rich resource of phytochemicals with ampelopsin (AMP, (2R,3R)-3,5,7-trihydroxy-2-(3,4,5-trihydroxyphenyl)-2,3-dihydrochromen-4-one) the major flavonoid. AMP is also called dihydromyricetin and has a similar structure to myricetin (3,5,7-trihydroxy-2-(3,4,5-trihydroxyphenyl)-4-chromenone), a naturally occurring flavonoid found in grapes, berries, fruits, vegetables, herbs and other plants with certain anti-cancer activities. As the major bioactive constituent of *Ampelopsis grossedentata,* AMP was shown to be mainly responsible for the reported biological activities, including hypoglycemic [2], anti-oxidative [3], [4] and hepatoprotective [4], [5] activities. AMP also enhanced the chemokinesis and chemotaxis effects of neutrophilic granulocytes and monocytes [6].

AMP was shown to possess certain anti-cancer activities. AMP inhibited the growth [7] and invasion of melanoma cells in vitro [8], [9], and inhibited metastasis of melanoma tumor in vivo [8], [9]. AMP inhibited the growth of lung tumors in vivo by inhibiting proliferation [10]. AMP had anti-angiogenesis activity by inhibiting the secretion of vascular endothelial growth factor (VEGF) and basic fibroblast growth factor (bFGF) from human hepatocellular carcinoma cells in vitro and also inhibited the growth of human hepatocellular carcinoma in mice [11]. AMP also reversed multidrug resistance in leukemia cells in vitro in part via decreasing the expression of p-glycoprotein [12]. On the other hand, the effect of AMP on the growth and progression of prostate cancer has not been studied.

The objectives of this study were to systematically evaluate AMP as a potential chemopreventive and therapeutic candidate against prostate cancer progression by using both in vitro and in vivo systems, and to elucidate the underlying cellular and molecular mechanisms of AMP actions. Our results provided experimental evidence to support the future development of AMP as an effective and safe candidate agent for the prevention and/or therapy of prostate cancer.

## Results

### Effects of AMP, total flavonoid extract (TFE) and myricetin on the proliferation of prostate cancer cell lines and normal prostate epithelial cells (PrEC) in vitro

**Figure 1:**
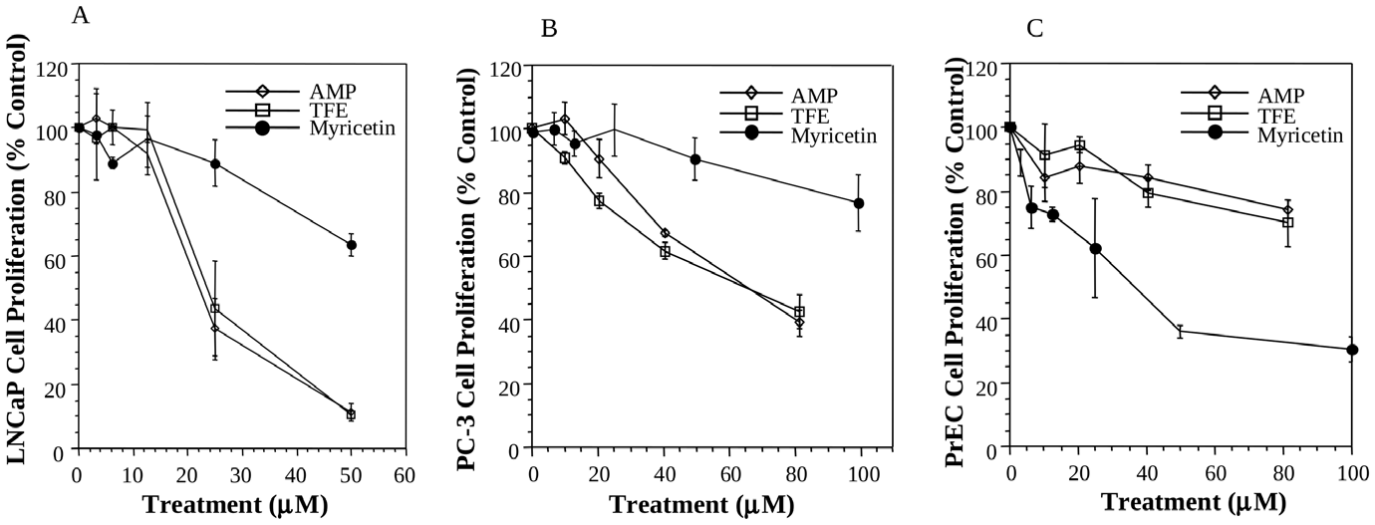
Effects of AMP, TFE and myricetin on the proliferation of human prostate cancer cells and normal prostate epithelial cells (PrEC) in vitro after 72 hr of treatments. A: The dose-dependent effects of AMP, TFE and myricetin on the proliferation of androgen-sensitive LNCaP cell line; B: The dose-dependent effects of AMP, TFE and myricetin on the proliferation of androgen-independent PC-3 cell line; C: The dose-dependent effects of AMP, TFE and myricetin on the proliferation of PrEC. Values are means±SEM of at least three independent experiments, each in triplicates.

We first compared the activity among AMP, TFE and myricetin in inhibiting the proliferation of prostate cancer cell lines and PrEC. The AMP purity analysis by high performance liquid chromatography (HPLC) showed that AMP purity was about 95%, and the representative HPLC chromatogram was shown in Fig. S1. TFE concentrations were calculated based on its AMP level, thus the difference of activity between TFE and AMP is attributed to other phytochemicals in TFE. As shown in Fig. 1 (A, B, C), AMP and TFE showed the similar activities in inhibiting the proliferation of prostate cancer cells or normal prostate cells, suggesting that AMP is the major bioactive flavonoid in TFE. AMP or TFE inhibited the proliferation of LNCaP cell line at the IC_50_'s about 25 µM (Fig. 1A), and that of PC-3 cell line at the IC_50_'s about 60 µM (Fig. 1B). On the other hand, AMP or TFE showed much less activity in inhibiting the proliferation of normal prostate epithelial cells than that of prostate cancer cell lines (Fig. 1C), suggesting that AMP or TFE may have moderate/minimal side effect.

On contrast, myricetin showed more potent activity in inhibiting the proliferation of PrEC than that of LNCaP or PC-3. Although myricetin inhibited the growth of prostate cancer cell lines at the IC_50_s >60 µM (Fig. 1A, 1B), it inhibited the growth PrEC cells at the IC_50_ about 35 µM (Fig 1C). Because of the potent anti-cancer activity and minimal side effect of AMP, it was used for further evaluation.

### Effects of AMP on cell cycle progression of prostate cancer cell lines in vitro

**Figure 2:**
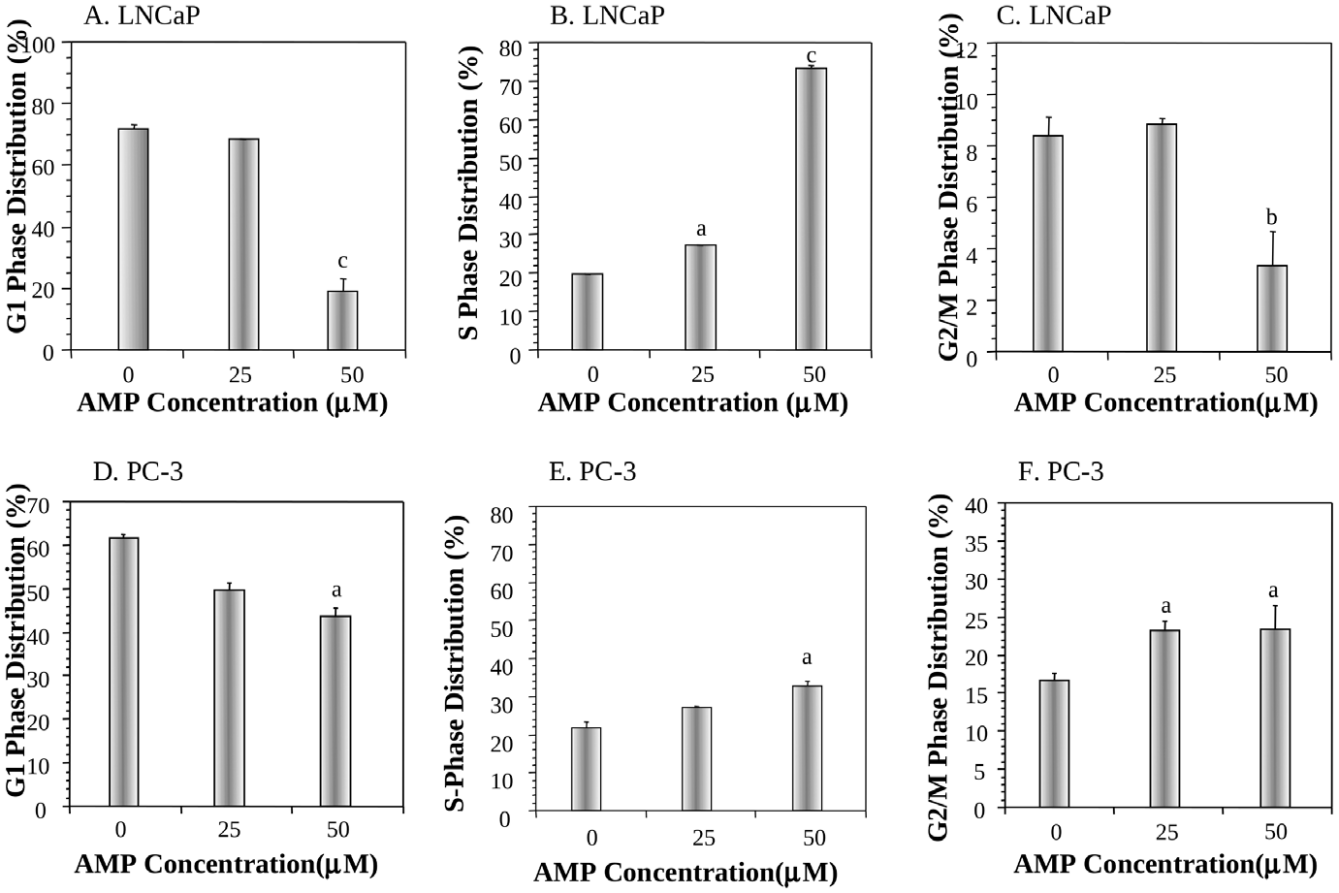
Effects of AMP on cell cycle progression of prostate cancer cell lines in vitro. A, B, and C: The dose-dependent effect of AMP on the distribution of LNCaP cells at G1 (A), S (B) and G2/M (C) phases; D, E, and F: The dose-dependent effect of AMP on the distribution of PC-3 cells at G1 (D), S (E) and G2/M (F) phases. Values are mean±SEM of three independent experiments, each in duplicates. Within the panel, the value with a letter is significantly different from that of the control, a, p<0.05; b, p<0.01; c, p<0.001.

AMP at 25 and 50 µM significantly increased the fraction of LNCaP cells at S phase from 20% to 28% (P<0.05) and 74% (P<0.001), respectively (Fig. 2B). On the other hand, AMP at 25 and 50 µM increased the fraction of PC-3 cells at S phase from 22% to 28% (P>0.05) and 34% (P<0.05), respectively (Fig. 2E), and at G2/M phases from 17% to 23% (P<0.05) and 24% (P<0.05), respectively (Fig. 2F).

**Figure 3:**
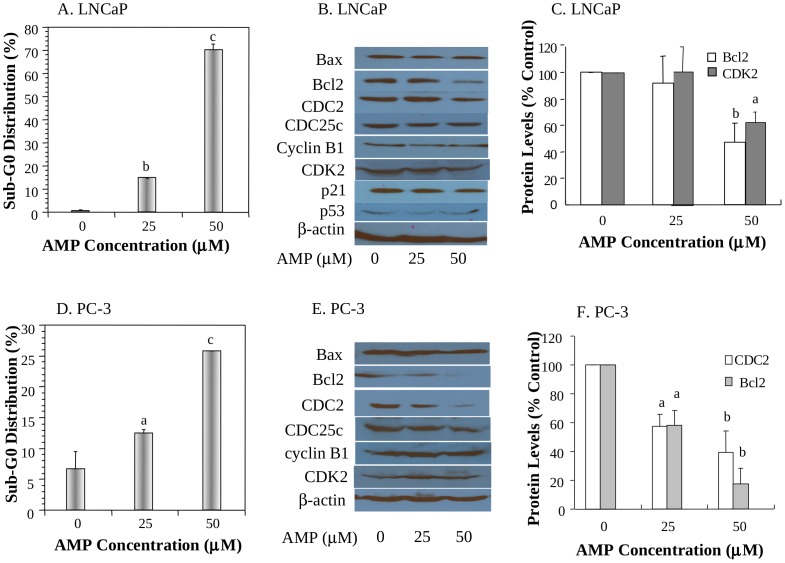
Effects of AMP on apoptosis of prostate cancer cells and on protein levels of biomarkers. A and D: The dose-dependent effect of AMP on the proportion of DNA fragmentation (sub-G_0_), a marker of apoptosis, in LNCaP (A) and PC-3 (D) cell lines; B and E: The representative Western blot images showing the effects of AMP on protein levels of cell cycle progression and apoptosis related biomarkers in LNCaP (B) and PC-3 (E) cell lines; C and F: Quantitation of significantly altered protein levels in LNCaP (C) and PC-3 (F) cell lines by densitometry after normalization to β-actin. The images for quantitation were from at least two independent experiments. Values are mean±SEM of three independent experiments, each in duplicates. Within the panel, the value with a letter is significantly different from that of the control, a, p<0.05; b, p<0.01; c, p<0.001.

Several cell cycle related biomarkers, such as cell division cycle 2 (CDC2), CDC25c, cyclin B1 and cyclin-dependent kinase 2 (CDK2), were determined by Western blot analysis. AMP significantly downregulated CDK2 protein level in LNCaP cells (Fig. 3B, 3C) and CDC2 protein level in PC-3 cells (Fig. 3E, 3F), but not other cell cycle related biomarkers.

### Effects of AMP on apoptosis induction of prostate cancer cell lines in vitro

AMP also significantly increased prostate cancer cell DNA fragmentation, a hallmark for apoptosis. AMP at 25 and 50 µM significantly induced DNA fragmentation of LNCaP cells by 15-fold (P<0.001) and 70-fold (P<0.001) respectively (Fig. 3A), and PC-3 cells by 86% (P<0.05) and 270% (P<0.001) respectively (Fig. 3D), compared with the corresponding controls. LNCaP cells were further treated with AMP at 25 and 50 µM and apoptosis related molecular biomarkers were determined by Western blot analysis. AMP induced apoptosis of LNCaP and PC-3 cells associated with downregulation of Bcl-2 (Fig. 3B, 3C, 3E, 3F).

The apoptosis induction effect of AMP on PC-3 cell line was further confirmed using the Annexin V-PI flow cytometry assay. AMP showed dose- and time- dependent effects on inducing PC-3 cell apoptosis (Fig. S2).

### Effects of AMP on migration and invasion of PC-3 cells and downregulation of CXCR4 protein level in vitro

**Figure 4:**
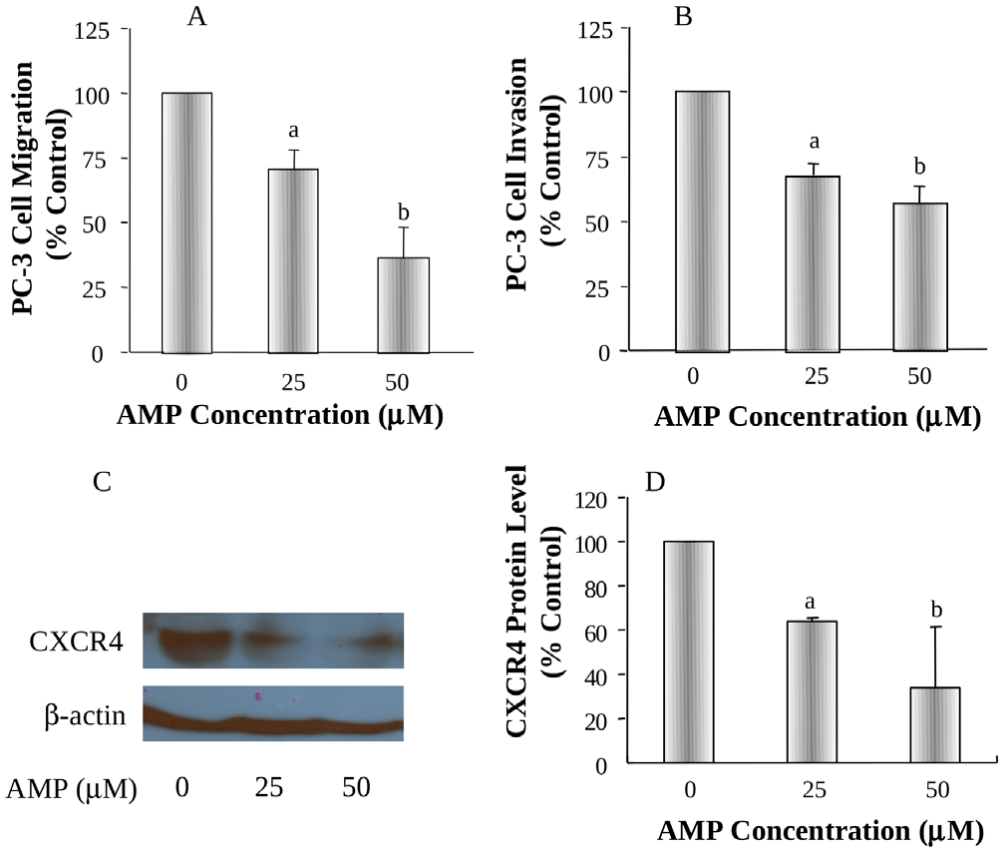
Effects of AMP on migration and invasion of PC-3 cells and CXCR4 protein levels in vitro. A: The dose-dependent effect of AMP on migration of PC-3 cells; B: The dose-dependent effect of AMP on invasion of PC-3 cells; C: The dose-dependent effect of AMP on CXCR4 protein levels in PC-3 cells; D: Quantitation of CXCR4 protein levels in PC-3 cells by densitometry after normalization to β-actin. Values are mean±SEM of three independent experiments, each in duplicate. Within the panel, the value with a letter is significantly different from that of the control, a, p<0.05; b, p<0.01; c, p<0.001.

PC-3 cell line was used to evaluate the effect of AMP on prostate cancer cell migration and invasion. AMP at 25 and 50 µM significantly inhibited PC-3 cell migration by 26% (P<0.05) and 63% (P<0.01), respectively (Fig. 4A), and invasion by 27% (P<0.05) and 45% (P<0.01), respectively (Fig. 4B). The inhibitory effect of AMP on PC-3 cell migration and invasion was associated with downregulation of CXCR4 protein level (Fig. 4C, 4D), an important biomarker for cancer cell invasion and metastasis. Under the experimental condition (16-hr treatment), AMP did not cause PC-3 cell cytotoxicity, as measured by the trypan blue assay (Fig. S3). It suggests that the observed anti-migration and anti-invasion activities of AMP are not secondarily due to its cytotixicity.

### Effects of AMP on the growth and metastasis of androgen-independent PC-3 prostate tumors and modulation of tumor cell proliferation, apoptosis and CXCR4 expression and tumor angiogenesis in mice

**Figure 5:**
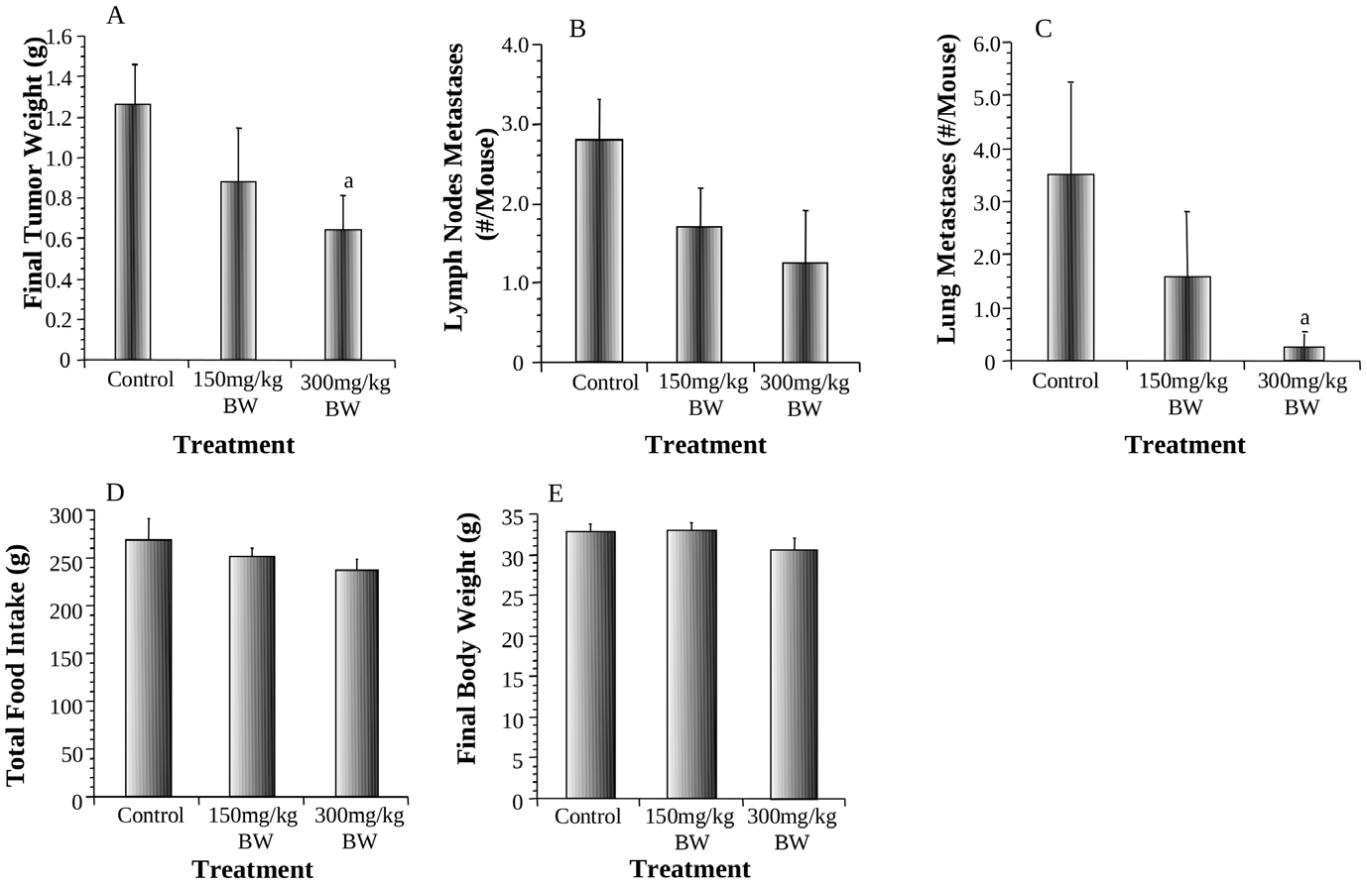
Effects of AMP treatment at 150 and 300 mg/kg BW on final PC-3 tumor weight (A), lymph node metastases (B), lung metastases (C), total food intake (D) and final body weight (E). Values are group mean±SEM (n = 8/group). Within the panel, the value with a letter is significantly different from that of the control, a, p<0.05.

An orthotopic PC-3 tumor animal model was used to evaluate the effect of AMP on preventing the growth and metastasis of prostate tumor. AMP inhibited both tumor growth and metastasis in vivo in a dose-dependent manner. AMP at 150 and 300 mg/kg BW reduced the final tumor weight by 28% (P>0.05) and 52% (P<0.05) respectively (Fig. 5A), inhibited lymph nodes metastases by 27% (P>0.05) and 43% (P>0.05) respectively (Fig. 5B), and inhibited lung metastases by 57% (P>0.05) and 86% (P<0.05) respectively (Fig. 5C). The representative images of lung metastases are shown in Fig. S4. On the other hand, AMP treatments did not significantly alter total food intake (Fig. 5D) or final body weight (Fig. 5E).

**Figure 6:**
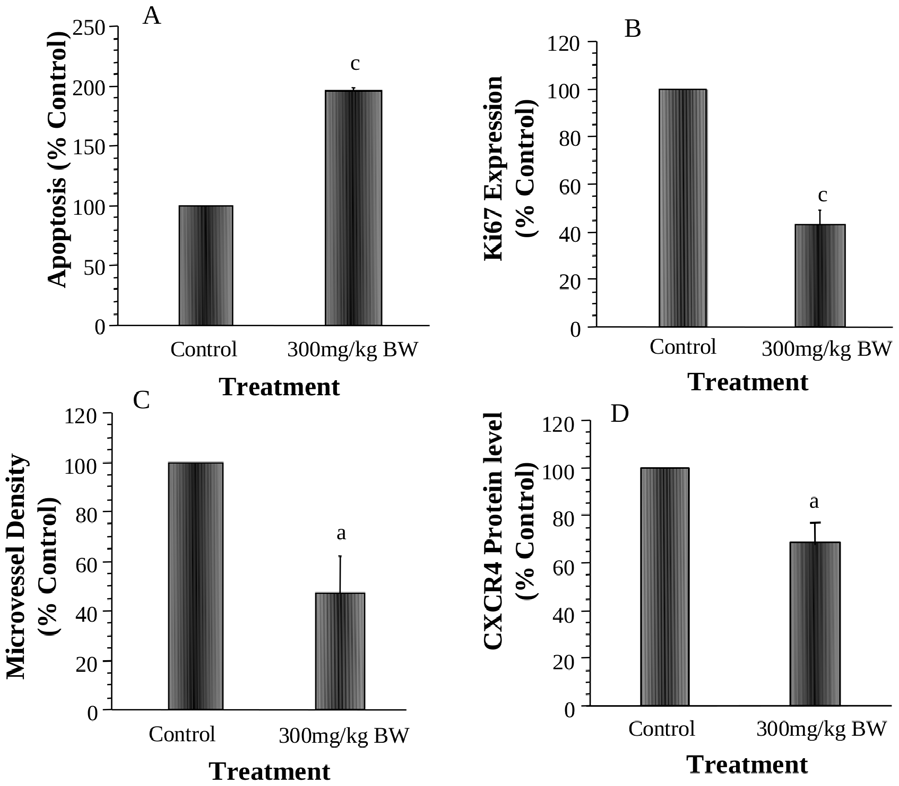
Effects of AMP treatment on PC-3 tumor cell apoptosis index measured by TUNEL assay (A), tumor cell proliferation measured by immunohistochemical staining of ki-67 (B), tumor microvessel density measured by Factor VIII staining (C) and tumor cell expression of CXCR4 measured by Western blot analysis (D). Values are group mean±SEM (n = 8/group). Within the panel, the value with a letter is significantly different from that of the control, a, p<0.05; c, p<0.001.

Analyses of cellular markers showed that AMP treatment significantly induced prostate cancer cell apoptosis by 200% (Fig. 6A, P<0.001), reduced prostate cancer cell proliferation by 60% (Fig. 6B, P<0.001) and inhibited prostate tumor angiogenesis by 58% (Fig. 6C, P<0.05). These results confirmed that AMP inhibited the growth of prostate tumors by inducing apoptosis, reducing proliferation, and inhibiting prostate tumor angiogenesis in vivo. The representative images of cellular biomarkers are shown in Fig. S5. The AMP treatment also reduced CXCR4 protein expression by 30% in prostate tumors (Fig. 6D, P<0.05).

## Discussion

In the present study, we found that AMP significantly inhibited the proliferation of prostate cancer cell lines via apoptosis induction associated with downregulation of Bcl2 expression, and suppressed prostate cancer cell migration and invasion associated with downregulation of CXCR4 expression in vitro. The animal study further confirmed that AMP significantly reduced the final tumor weight associated with inducing prostate cancer cell apoptosis, inhibiting prostate cancer cell proliferation and reducing prostate tumor angiogenesis, and significantly inhibited lung metastases. On the other hand, AMP at effective doses did not show significant adverse effect on food intake or body weight. This is the first study, to the best of our knowledge, that demonstrated the inhibitory effect of AMP on the growth and metastasis of prostate cancer in vitro and in a clinically relevant orthotopic tumor model.

Cellular mechanism studies showed that AMP inhibited the proliferation of prostate cancer cells. Interestingly, AMP arrested LNCaP cells at S phase (Fig. 2B) in part via down regulation of CDK2 (Fig. 3B, 3C), a biomarker essential for the G1/S transition, whereas it arrested PC-3 cells at S and G2/M phases (Fig. 2E, 2F) associated with downregulation of CDC2 (Fig. 3E, 3F). CDC2, also known as CDK1 (cyclin dependent kinase 1), plays an important role during the cell cycle progress. CDC2 usually combines with cyclin B and regulates the S and G2/M phase progression. CDC2 has been considered as an essential molecular target for design of therapeutic anti-cancer drugs [13]. Down-regulation of CDC2 may provide an important molecular mechanism that AMP arrests cell cycle progression of PC-3 cells at S and G2/M phases. Analysis of in vivo tumor samples also confirmed that AMP inhibited PC-3 tumor growth associated with inhibition of tumor cell proliferation (Fig. 6B).

Induction of prostate cancer cell apoptosis is also an important mechanism by which AMP inhibit the growth of prostate cancer. AMP induced apoptosis of prostate cancer cells in vitro (Fig. 3A, 3D) and in vivo (Fig. 6A). Further investigation showed that induction of apoptosis was associated with down regulation of bcl-2 protein levels (Fig. 3C, 3F). Bcl-2 is an important regulator of apoptosis. Expression of bcl-2 is more frequent in high-grade tumors and metastases than in lower-grade and nonmetastatic tumors [14], [15]. Apoptosis resistance is associated with higher levels of bcl-2 [15], [16], and downregulation of bcl-2 sensitizes prostate cancer cells to undergo apoptosis [17]. Therefore, downregulation of bcl-2 could represent an important molecular mechanism by which AMP induces prostate cancer apoptosis.

Angiogenesis is a critical step in tumor growth [18]. The growth of all solid tumors depends on angiogenesis and suppression of tumor blood vessel offers a new option for the prevention and treatment of cancer [19]. Previous studies showed that AMP had anti-angiogenesis activity by inhibiting the secretion of pro-angiogenic factor VEGF and bFGF from human hepatocellular carcinoma cells in vitro [11]. In this study, we demonstrated that AMP inhibited the growth of PC-3 prostate tumor associated with inhibition of tumor angiogenesis (Fig. 6C). Although we did not measure the VEGF and bFGF levels in vivo, our results provided suggestive experimental evidence to support that one of the mechanisms by which AMP inhibits tumor growth is via inhibiting angiogenesis. Further investigation is needed to determine the underlying molecular mechanisms that AMP inhibits tumor angiogenesis.

Previous studies have shown that AMP inhibited the in vitro invasion and in vivo metastasis ability of B16 melanoma [8]. However, it has not been studied if AMP had anti-metastasis activity in prostate cancer. Our results demonstrated that AMP had potent activities in inhibiting migration and invasion of prostate cancer cells in vitro and prostate cancer metastasis in the orthotopic prostate tumor animal model associated with downregulation of CXCR4 expression. CXCR4 is involved in several diseases such as angiogenesis, metabolic and neurological disorders, rheumatoid arthritis and in different forms of metastatic cancer. CXCR4 is a critical factor in the invasiveness and proliferation of breast cancer cells [20], [21], [22] and plays a pivotal role for the growth of malignant neuronal and glial tumors [23]. CXCR4 inhibitors are suggested for the treatment of different forms of metastatic cancer [24], [25], [26]. AMP is shown to be a small molecule CXCR4 antagonist [27], [28]. Although we did not study if CXCR4 was a direct molecular target for AMP, our studies suggest that one of the molecular mechanisms by which AMP inhibits prostate cancer metastasis may be via downregulation of CXCR4 expression and function. Further investigation in applying AMP and/or its derivatives as novel anti-metastasis agents for delaying and/or preventing cancer metastasis could have significant impacts on prevention and therapy of cancer.

In conclusion, the results from this study provided critically important experimental evidence to suggest that AMP may be a novel efficacious and safe candidate agent to inhibit the growth and metastasis of prostate cancer.

## Materials and Methods

### Ethics statement

All procedures with animals were reviewed and approved by the Institutional Animal Care and Use Committee at Beth Israel Deaconess Medical Center with the Guide for the Care and Use of Laboratory Animals.

### TFE, AMP and myricetin

A proprietary extraction procedure was applied to extract TFE from *Ampelopsis grossedentata* with AMP content at approximately 80%. AMP was purified from TFE by preparative HPLC; and the purity was verified by analytical reverse phase HPLC. Myricetin was purchased from LKT Laboratories (St. Paul, MN). HPLC analysis was used to verify the quality of the materials. All materials were dissolved in dimethyl sulfoxide (DMSO) for cell culture studies.

### Culture of prostate cancer cells, normal prostate epithelial cells and endothelial cells

Two human prostate cancer cell lines, androgen-sensitive LNCaP and androgen-independent PC-3 (American Type Culture Collection, Bethesda, MD) were used for the in vitro experiments. Human prostate cancer cell lines were maintained as monolayer cultures in DMEM medium supplemented with 10% fetal bovine serum, 2 µmol L-glutamine/mL, 100 U penicillin/mL and 100 µg streptomycin/mL in a 95% air, 5% CO_2,_ and water-saturated atmosphere. PrEC cells were purchased from Lonza (Walkersville, MD) and cultured in PrEGM plus EGM-2 singlequotes (Lonza, Walkersville, MD) in a humidified atmosphere of 95% air and 5% CO_2_.

### Cell growth

The effect of AMP, TFE and myricetin on the cytotoxicity of prostate cancer cells or PrEC was determined using the Cell Titer 96 Aqueous Cell Proliferation Assay (Promega, Madison, WI) as we previously described [29]. The assay determines the number of viable cells in proliferation, cytotoxicity and chemosensitivity. All assays were independently repeated at least thrice, each in triplicate, and results were confirmed by direct cell counting using a hemocytometer.

### Analysis of prostate cancer cell cycle progression and DNA fragmentation

The effect of AMP treatment on cell cycle distribution of prostate cancer cell lines was determined by flow cytometry following the described procedures [30]. Cells treated with different concentration of AMP were harvested, stained with propidium iodide (at a final concentration of 50 µg/ml) and RNase (at a final concentration of 50 µg/ml), and incubated at 37°C for 30 min. Stained cells were then analyzed by FACScan (Becton Dickinson, Immunocytometry Systems, Mountview, CA) for cell cycle distribution and fragmented DNA. The experiments were independently repeated at least thrice, each in duplicate.

### Annexin V and PI staining for apoptosis detection

The effect of AMP on the apoptosis of PC-3 cells was further determined by Annexin V-PI apoptosis detection kit (Chemicon International Inc, Billerica, MA) following the instruction of kit. Briefly, treated PC-3 cells were resuspended in Annexin V solution and incubated at room temperature for 15 min, PI was then added for another 5-min incubation in the dark. Apoptotic cells were analyzed by flow cytometry (Becton Dickinson, Immunocytometry Systems, Mountview, CA). The experiments were independently performed at least twice, each in duplicates.

### Cancer cell migration and invasion assays

A suspension of PC3 cells (6000 cells for migration and 30000 cells for invasion) in 250 µL of serum-free DMEM with AMP or the vehicle (DMSO) was loaded into a fibronectin-coated insert for migration assay or a matrigel-coated insert for invasion assay (Biocoat, 24-well plate, 8 µm, Becton Dickinson). Each culture insert was set into a well containing 750 µL of DMEM with 5% FBS. After incubation for 16 hrs at 37°C, cells on the top of inserts were removed carefully using a cotton tip, and the cells on the underside of the insert membrane were stained with Diff-Quick Stain solutions (Dade Behring Inc., Newark, DE) and the images were captured. Cells were counted under a microscope. All assays were independently performed at least thrice, each in triplicate.

To confirm that the anti-migration and anti-invasion activities of AMP were not secondarily due to its cytotoxicity to PC-3 cells, PC-3 cells were treated with AMP (0, 25, and 50 µM) for 16 hr, and the cell numbers were measured by trypan blue exclusion assay. The experiment was independently performed at least thrice, each in duplicate.

### Western blot analysis

For the in vitro study, cells were treated with different concentrations of AMP, cell lysates were prepared, and protein levels were determined by Western blot analysis following the procedures we described previously [31]. The primary antibodies used in Western blot against human antigens were as follows: cyclin B1 (1∶200), cdc2 (1∶500) and bax (1∶500) (Oncogene Research Products, Boston, MA), bcl-2 (1∶100), p21 (1∶200) and p53 (1∶500) (Santa Cruz Biotechnology, Santa Cruz, CA), CDK2 (1∶1,000) (Selleck Chemicals, Houston, TX), CXCR4 (1∶200) (R&D systems, Inc, USA), and β-actin (1∶10,000) (Merck Co., Darmstadt, Germany). For the in vivo study, tumor samples in both the control and AMP-treated groups were collected for Western blot analysis of CXCR4 protein level.

### Animal study

The chemopreventive intervention activity of AMP on the growth and metastasis of PC-3 tumors was determined in an orthotopic PC-3 tumor animal model. Male severe combined immune-deficient mice (6–8-week old) were purchased from Taconic (Germantown, NY), and housed at the animal facility of Beth Israel Deaconess Medical Center in a pathogen-free environment equipped with laminar flow hoods and standard vinyl cages with air filters. After one week of acclimatization and adaptation to the AIN-93 diet, mice were randomly assigned into one of the following three experimental groups (in each group, n = 8) and pretreated via gavage daily for 2 weeks: (i) Control: the vehicle (100 µL corn oil); (ii) AMP at 150 mg/kg body weight (BW); and (iii) AMP at 300 mg/kg BW. Each mouse was then implanted orthotopically with PC-3 cells (2×10^6^ cells/50 µL) and continued on the corresponding treatments throughout the experiment. Food intake and body weight were measured weekly. At the end of the experiment (8 weeks after PC-3 cell implantation), the mice were sacrificed; primary tumors were excised and weighed. A tumor slice from each primary tumor tissue was carefully dissected and fixed in 10% buffer-neutralized formalin, paraffin-embedded, and sectioned at 4 µm thickness for immunohistochemistry. All procedures with animals were reviewed and approved by the Institutional Animal Care and Use Committee at Beth Israel Deaconess Medical Center with the Guide for the Care and Use of Laboratory Animals [32]. The animal study was repeated.

### In situ detection of apoptotic index

Apoptotic cells were determined by a terminal deoxynucleotidyl transferase-mediated deoxyuridine triphosphate-biotin nick end labeling (TUNEL) assay (Chemicon International Inc, Billerica, MA) following our described protocols [30], [33], [34]. Six representative areas of each section without necrosis were selected and both apoptotic cells and total nuclei cells were counted under a light microscope at 400× magnification. The apoptotic index was expressed as the percentage of positive apoptotic tumor cells to total tumor cells, and the effect of treatment on apoptosis was expressed as the percentage to the control.

### Immunohistochemical determination of tumor cell proliferation

The proliferation index was evaluated by calculating the proportion of cells with Ki-67 staining, following the procedures in the laboratory [31]. Both Ki-67-positive proliferating cells and total tumor cells were counted in six non-necrotic areas of each section using light microscope at 400× magnification. The proliferation index was calculated as the percentage of Ki-67-positive tumor cells to total tumor cells, and the effect of treatment on proliferation was expressed as the percentage to the control.

### Immunohistochemical detection of microvessel density (MVD)

MVD was used as a marker for tumor angiogenesis and quantified by immunohistochemical staining of Factor VIII following our previously described method [30], [33], [34]. MVD was calculated by counting microvessels on 200× fields under light microscopy at six representative sites without necrosis of each section, and the effect of treatment on angiogenesis was expressed as the percentage to the control.

### Statistical analysis

Results were expressed as group means±SEM and analyzed for statistical significance by analysis of variance [35] followed by Fisher's protected least-significant difference based on two-side comparisons among experimental groups using Statview 5.0 program (SAS Institute, Inc., Cary, NC). A *P*<0.05 was considered statistically significant.

## Supporting Information

Figure S1 **The representative chromatogram showing the purity of AMP by analytical reverse phase HPLC.** HPLC experimental conditions were: Instrument: Waters 2695 with auto-sampler and the photo diode array (PDA) detector; HPLC column: Phenomenox C-18 (5 µm in inner diameter, 10×250 mm); Mobile phases: A (H_2_O with 0.1% acetic acid) and B (acetonitrile); Gradient condition: 0–30 min, 5% B to 35% B; 30–35 min, 35% B to 95% B; 35–40 min, 95% B to 5% B; 40–45 min, 5% B; Flow rate: 1 ml/min.
(TIF)

Figure S2 **Representative FACS histograms showing the time-dependent effect of AMP treatments (0, 20, and 50 µM) on apoptosis of PC-3 cells, as measured by the Annexin-PI flow cytometry assay.**
(TIF)

Figure S3 **The effect of AMP on PC-3 cell cytotoxicity, as measured by the trypan blue exclusion assay.** The cells were treated with AMP at different concentrations for 16 hr, the same time used for migration and invasion assays. Values are mean±SEM of three independent experiments, each in duplicates. The values are statistically insignificant among groups.
(TIF)

Figure S4 **The representative H&E images showing the inhibitory effect of AMP at 150 mg/kg body weight (BW) (B) and 300 mg/kg BW (C) on lung metastases, as compared with the control group (A).**
(TIF)

Figure S5 **The representative images showing the effects of AMP (300 mg/kg BW) on the expression of cellular biomarkers of proliferation, as measured by ki-67 staining (A), apoptosis as measured by TUNEL assay (B) and angiogenesis, as measured by Factor VIII staining for microvessel density (C).**
(TIF)
