# Supplementary figures and images for: Flavonoid Ampelopsin Inhibits the Growth and Metastasis of Prostate Cancer In Vitro and in Mice
Source: PLoS One. 2012 Jun 5;7(6):e38802. doi: 10.1371/journal.pone.0038802 (PMC3367907; doi:10.1371/journal.pone.0038802)

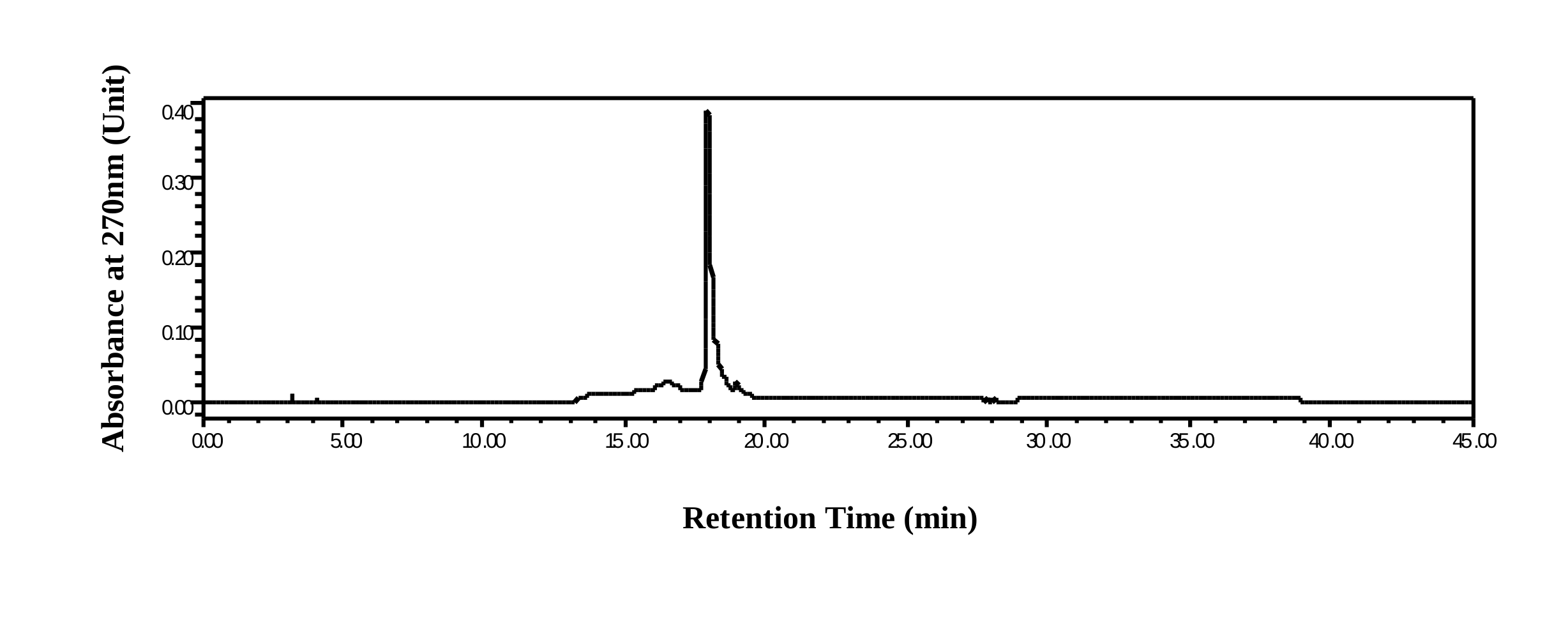

Supplement: Figure S1 — The representative chromatogram showing the purity of AMP by analytical reverse phase HPLC. HPLC experimental conditions were: Instrument: Waters 2695 with auto-sampler and the photo diode array (PDA) detector; HPLC column: Phenomenox C-18 (5 µm in inner diameter, 10×250 mm); Mobile phases: A (H2O with 0.1% acetic acid) and B (acetonitrile); Gradient condition: 0–30 min, 5% B to 35% B; 30–35 min, 35% B to 95% B; 35–40 min, 95% B to 5% B; 40–45 min, 5% B; Flow rate: 1 ml/min. (TIF) [file pone.0038802.s001.tif]

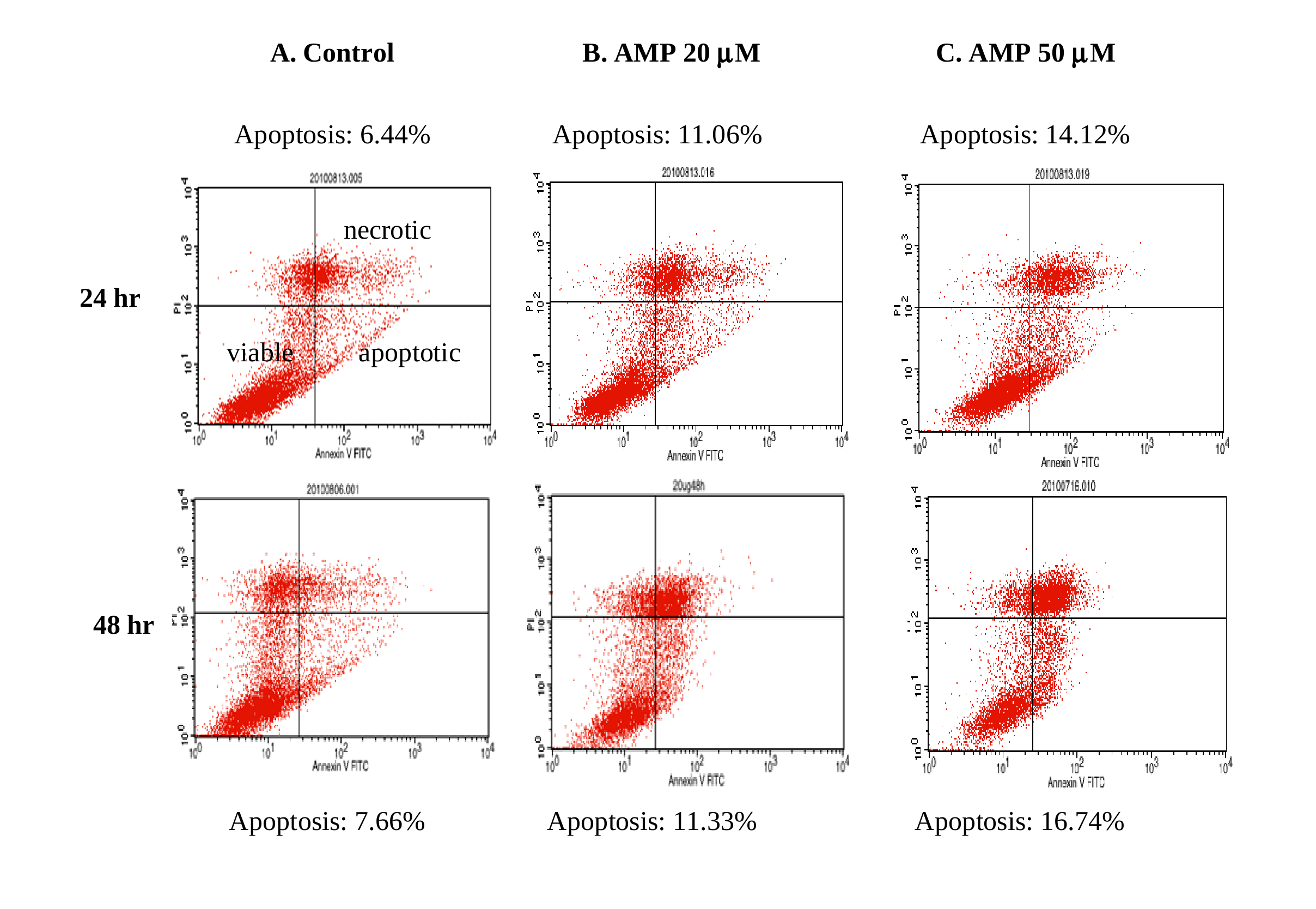

Supplement: Figure S2 — Representative FACS histograms showing the time-dependent effect of AMP treatments (0, 20, and 50 µM) on apoptosis of PC-3 cells, as measured by the Annexin-PI flow cytometry assay. (TIF) [file pone.0038802.s002.tif]

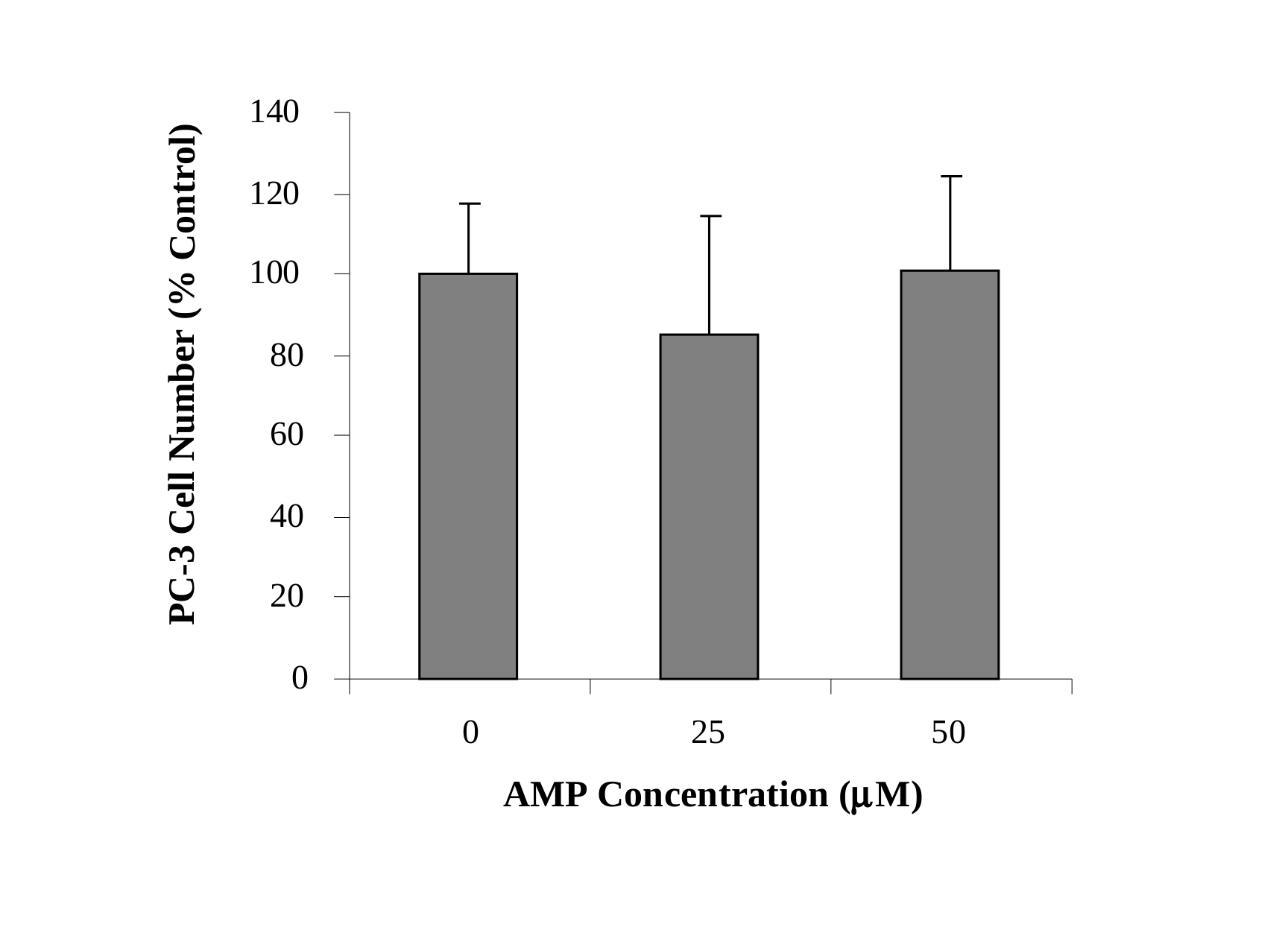

Supplement: Figure S3 — The effect of AMP on PC-3 cell cytotoxicity, as measured by the trypan blue exclusion assay. The cells were treated with AMP at different concentrations for 16 hr, the same time used for migration and invasion assays. Values are mean±SEM of three independent experiments, each in duplicates. The values are statistically insignificant among groups. (TIF) [file pone.0038802.s003.tif]

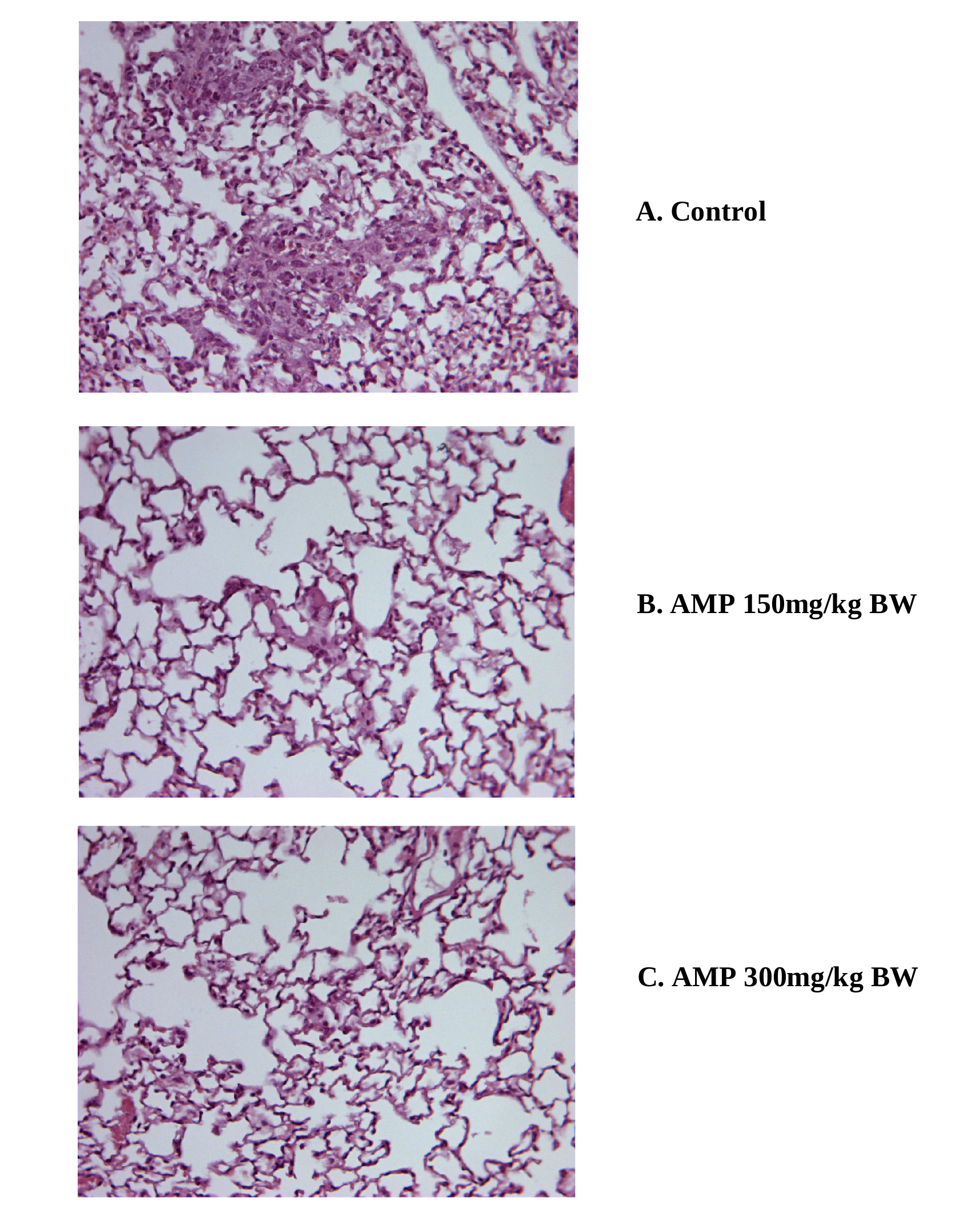

Supplement: Figure S4 — The representative H&E images showing the inhibitory effect of AMP at 150 mg/kg body weight (BW) (B) and 300 mg/kg BW (C) on lung metastases, as compared with the control group (A). (TIF) [file pone.0038802.s004.tif]

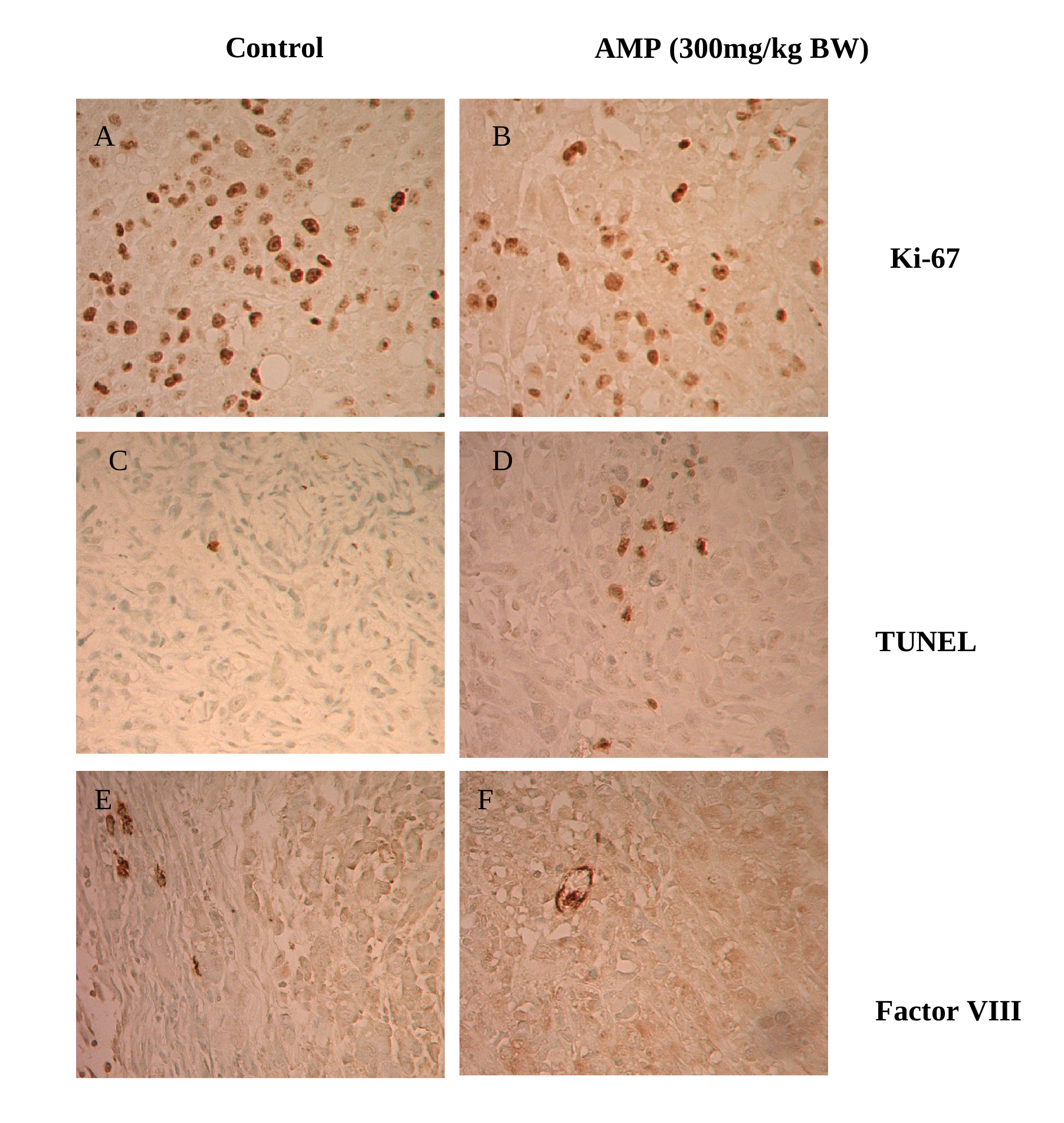

Supplement: Figure S5 — The representative images showing the effects of AMP (300 mg/kg BW) on the expression of cellular biomarkers of proliferation, as measured by ki-67 staining (A), apoptosis as measured by TUNEL assay (B) and angiogenesis, as measured by Factor VIII staining for microvessel density (C). (TIF) [file pone.0038802.s005.tif]
